# Preliminary reference intervals and the impact of citrate storage time for thrombelastography in cats including delta and the velocity curve

**DOI:** 10.1186/s12917-017-1278-y

**Published:** 2017-11-29

**Authors:** Carolin Engelen, Andreas Moritz, Franziska Barthel, Natali Bauer

**Affiliations:** 1 Department of Veterinary Clinical Sciences, Clinical Pathophysiology and Clinical Pathology, Justus-Liebig University Giessen, Frankfurterstraße 126, 35392 Giessen, Germany; 2 Bayer Animal Health GmbH, Alfred-Nobel-Straße 50, 40789 Monheim am Rhein, Germany

**Keywords:** Feline, Hypercoagulable, TEG, Velocity curve

## Abstract

**Background:**

Thrombelastography is a useful tool in assessment of hemostasis. Beside the traditional variables, the velocity curve and the variable delta have lately earned attention. The velocity curve provides knowledge about the speed of clot formation including information about thrombin generation. Delta, which only reflects enzymatic coagulation, allows the determination of the origin of hypercoagulability when compared to clot rigidity, a variable that reflects both platelet and enzymatic activity. The aim was to establish preliminary reference intervals for feline thrombelastography including the velocity curve variables and delta obtained after 60 min of storage including the assessment of coefficients of variation. Furthermore, the effect of citrate storage time (30 versus 60 min) on feline thrombelastography will be determined.

**Results:**

Prolonged storage times significantly reduced reaction (R) (*P* = 0.019) and clotting (K) (*P* = 0.008) times, split point (SP) (P = 0.019) and time to maximum rate of thrombus generation (TMRTG) (*P* = 0.023) values whereas maximum rate of thrombus generation (MRTG) significantly increased (*P* = 0.040). Preliminary reference intervals: R (min): 2.7–18.1; K (min): 0.8–3.9; alpha (°): 27.6–75.2; maximum amplitude (mm): 18.5–62.5; clot rigidity (dyn/cm^2^): 1.2–8.2; coagulation index: −4.6 – 2.6; SP (min): 2.4–15.4; delta (min): 0.3–3.1; thrombus generation (mm/min): 255.3–751.2; MRTG (mm/min): 4.0–19.3; TMRTG (min): 3.5–22.0; maximum rate of lysis (mm/min): 0.0–4.7 and time to maximum rate of lysis (min): 0.4–55.8.

**Conclusion:**

Storage for 60 versus 30 min induces hypercoagulable tracings including the velocity curve, some of which variables (MRTG, TMRTG) might function as sensitive markers for changes in the coagulation activity. Because of the impact of citrate storage time on thrombelastography, reference intervals have to be established using a specific and constant storage time in each laboratory.

## Background

**Fig. 1:**
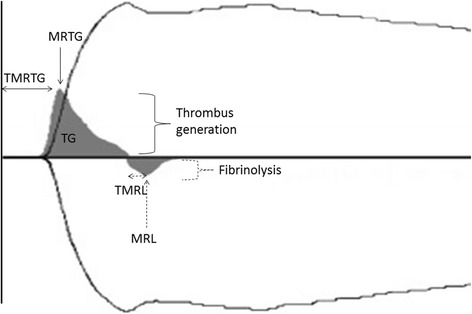
Feline thrombelastography (TEG) showing the conventional tracing (black line) with superimposed velocity curve (gray solid curve)**.** The gray curve above the horizontal axis reflects thrombus generation while the gray curve below represents fibrinolysis. Measurements that can be estimated include TMRTG (time to maximum rate of thrombus generation; min), MRTG (maximum rate of thrombus generation; mm/min), TG (thrombus generation; mm/min) from the area under the curve (AUC), TMRL (time to maximum rate of lysis; mm/min) and MRL (maximum rate of lysis; min)

**Fig. 2:**
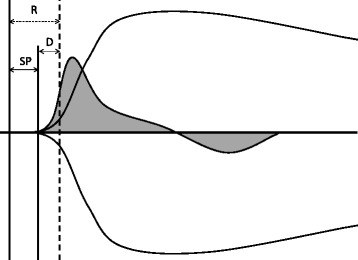
Schematic representation of the variable delta (D; min; black arrow), which can be calculated from the difference between the reaction time (R; min; dotted arrow), i.e., the time from initiation of coagulation until the amplitude of the conventional TEG tracing reaches 2 mm and the split point (SP; min; longer black arrow), i.e., the time from starting the coagulation process until divergence of the TEG tracings from the zero line. The delta value = R - SP

Thrombelastography (TEG) is a global coagulation test that enables assessment of hemostatic function in whole blood combining plasma and cellular components of hemostasis [1–3]. Recently, the global integrity of the complex process of blood coagulation is preferably assessed by measurement of thrombin generation [4], which can be evaluated using the TEG velocity curve (VC) [4] thus gaining new attention in human and veterinary medicine. The VC is generated from the mathematical first derivative of TEG values and dynamically reflects the velocity of clot formation (positive curve above the horizontal axis) and clot dissolution (negative curve below the horizontal axis) [5, 6] as illustrated in Fig. 1. Variables generated from the curve of clot formation include the maximum rate of thrombus generation (MRTG) represented by the peak of this curve [5]. In human medicine, MRTG has been shown to increase exponentially compared with the non-exponential and thus less pronounced increase of TEG alpha as a traditional marker of clot formation, thus allowing a more precise evaluation of clot kinetics [7]. Other variables reflecting clot formation derived from the velocity curve include the time to maximum rate of thrombus generation (TMRTG), defined as the distance on the x-axis from zero to the peak of the curve, and the thrombus generation (TG), which is calculated from the area under the curve and represents an indirect measurement of clot strength [5]. TMRTG is also believed to be a good marker of thrombin generation in humans and shows a linear correlation with the TEG parameter delta [6]. Delta is easily obtained from the TEG tracing by calculating the difference between two parameters R and SP of the traditional TEG curve [6] as shown in Fig. 2. Delta represents the greatest clot growth depending on peak thrombin generation, thus allowing the assessment of thrombin generation [6]. In people, the variable TG has been shown to correlate with thrombin-antithrombin complex values which serve as a marker of thrombin generation [4]. Values that can be obtained from the curve below the horizontal axis reflect fibrinolysis and include the maximum rate of lysis (MRL) represented by the negative peak of this curve and the time to maximum rate of lysis (TMRL), which is the represented on the x-axis from the start of the fibrinolysis curve to its negative peak [5].

The TEG software provides two modes of calculating the VC variables: first, VC variables can be calculated on the basis of the maximum amplitude (MA), a traditional TEG variable. Second, VC variables can be derived from the clot rigidity (G), which itself is calculated from MA and represents the global coagulation activity. Like MA, G is dependent on factor XIII activity, concentration of fibrinogen and thrombin, the platelet count and function, and hematocrit [8].

Both versions (MA and G version) of calculation result in slightly different curves and subsequently different results. Furthermore, the variable names differ: G version variables have a “G” appended to the end of the name, i.e. MRTGG instead of MRTG [9].

From the clinical point of view, the TEG gained special interest for the evaluation of hypercoagulability [10], whereby it has an exceptional ability to detect individuals affected by or at risk for a thromboembolic event [10, 11]. In human medicine, hypercoagulable states measured by rapid thrombelastography have already been shown to be a good predictor of thromboembolic events [12]. To help differentiating the contribution of enzymatic activity (reflecting secondary hemostasis) and platelet reactivity (reflecting primary hemostasis) in hypercoagulable states, the novel variable delta which represents the enzymatic activity has been introduced [6]. In correlation with the variable G (reflecting both primary and secondary hemostasis), hypercoagulable states can be classified as predominantly caused by platelet hypercoagulability if the patients show a hypercoagulable G value and a normal delta [6]. Neither delta nor the VC variables have yet been evaluated in cats.

For interpretation of TEG results, pre-analytical factors such as the sample material and the time between sampling and TEG analysis have to be taken into account. Originally designed to be run with fresh whole blood [3, 13], TEG analysis in veterinary medicine is mostly done using re-calcified citrated whole blood [10, 14]. Because citrate does not entirely prevent thrombin generation since the generation of factor XIIa (via contact activation) is not dependent on calcium, contact activation is not inhibited in citrated samples and continues during storage, resulting in a certain degree of thrombin generation [8, 13]. Consequently, the citrate storage time has an impact on TEG results [13], as demonstrated previously for people, dogs and horses [3, 15–18]. The impact of citrate storage time on feline TEG is largely unknown. Even if a storage time of 30 min has been recommended [13] and has mostly been used in previous investigations in cats [19–24], a storage time of 60 min might be preferable in some clinical settings especially if samples are used for a variety of different coagulation tests.

Thus, one aim of this study was to establish preliminary reference intervals (RIs) including assessment of coefficients of variation (CVs) for traditional and novel feline TEG parameters using 60 min incubation. The second aim was to determine the effect of citrate storage time (30 versus 60 min) on TEG results which, to our knowledge, has not yet been done before in cats.

## Methods

### Study design

This prospective study was performed September 2015 and ethical approval has been given by the State Agency for Nature, Environment and Consumer Protection of North Rhine-Westphalia (LANUV NRW, reference number: 84–02.05.20.13.090, internal number: 200a176/232). Blood samples were collected from 21 clinically healthy adult domestic shorthair cats from Bayer Animal Health GmbH (Leverkusen, Germany) including 12 female and 9 male cats with a mean age of 12.3 months (range 12–13 months). The cats have been obtained from one breeder (Liberty Research, Waverly, US) and were kept under defined housing conditions in groups according to their gender. A commercially available food was fed (Josera Léger, Adult light, Kleinheubach, Germany) and water was available ad libitum.

Initial health status of the cats was determined through a routine physical examination. The blood was collected from the cephalic vein using a 21G needle (0.8 × 16.0 mm, Neolus needle, Terumo, Eschborn, Germany) and dropped into two 1.4-ml 3.2% sodium citrate tubes (S-Monovette®, 1.4-ml, 9NC, Sarstedt, Wedel, Germany) which were properly filled ensuring an ratio of blood to anticoagulant of 9:1. In a randomized order, one tube of citrated whole blood was used for TEG analysis. From the other tube, citrated plasma for a routine coagulation profile including thrombin time (TT), prothrombin time (PT), activated partial thromboplastin time (aPTT), fibrinogen concentration and D-Dimers was obtained within 20 min after sampling by centrifugation (Eppendorf 58 10 R, Eppendorf AG, Hamburg, Germany) for 10 min. The plasma was then transferred into plain tubes (Eppendorf Safe-Lock Tubes, Eppendorf AG, Hamburg, Germany) and frozen at −80 °C. For coagulation analyses, frozen samples were shipped on dry ice to the department of Veterinary Clinical Sciences, Clinical Pathophysiology and Clinical Pathology (Justus-Liebig University, Giessen, Germany), and stored at −80 °C until measurements were performed as batch on the automated coagulation analyzer STA Compact ® (Stago Germany, Düsseldorf, Germany) within a year after sample collection as described previously for dogs [25].

One week prior to the study, a complete blood count (CBC) measured with the automated hematology analyzer ADVIA® 120 Hematology System (Siemens HealthCare GmbH, Erlangen, Germany; software version: no. 1) which has been evaluated previously for cats [26] and a biochemistry profile (IDEXX Catalyst Dx®, IDEXX GmbH, Ludwigsburg, Germany) were performed. Results were compared with RIs provided by the manufacturer of the clinical chemistry analyzer. Cats were included in our study when they were considered healthy based on the physical examination and the absence of clinically relevant abnormalities of the hematological and blood chemical examination.

The hematocrit and platelet count were reevaluated from citrated blood at the day of blood sampling for TEG analysis. Results were corrected by the factor 1.1 to take the dilution of the sample with citrate into consideration. The hematocrit value was evaluated with the microhematocrit method after centrifugation of 5 min at 31,689 G using a microhematocrit-centrifuge (“Haematokrit 24”, Hettich, Tuttlingen, Germany). The platelet count was estimated semiquantitatively by counting platelets in the monolayer of a May-Grünwald-Giemsa stained blood smear, whereby the mean of 10 high power fields (100× objective) was calculated and multiplied by a factor of 20 as previously recommended [27] to obtain the approximate platelet count (×10^3^/μl). Thrombocytopenia was defined as values below 200 (×10^3^/μl) [26]. When moderate to marked platelet clumping was seen, the assessed count was judged falsely low. Moderate to marked platelet clumping was defined as a high number of platelets within clumps that did not allow accurate counting of individual platelets [27]. As an addition to the size of clumps, the number of clumps was used to further classify the platelet clumping as low, moderate or high grade clumping. According to this classification, 13 cats were categorized as having low grade, 2 cats as moderate and 5 cats as having high grade platelet clumping.

### TEG analysis

The analyses were always performed by the same person (CE) using three computerized TEG 5000 analyzers (Thrombelastograph, Haemonetics Corporation, Braintree, MA, USA). TEG analyses were done with recalcified whole blood stored at room temperature and according to the manufacturer recommendations. Briefly, standard TEG cups (Haemoscope Corporation) were placed in the 37 °C prewarmed instrument holder and filled with 20 μl of 0.2 M calcium chloride. After specific storage times of either 30 and/or 60 min respectively, 340 μl citrated whole blood was added and analysis was started. Each TEG analysis was performed for approximately 60 min and an electric internal quality control (e-test) was performed at least three times a day.

Blood from the first 10/21 cats was stored for 60 min before TEG analysis, which was run in duplicates using blood from the same tube to assess the variance. TEG assays for the remaining 11/21 cats were performed with blood taken from the same tubes after 30 and 60 min of storage time respectively to assess sample stability. One of these 11 cats had to be excluded from the study since TEG analysis only showed flat lines.

Evaluated TEG variables include the traditional variables: reaction time (R), clotting time (K), alpha, maximum amplitude (MA), clot rigidity (G), coagulation index (CI) and clot lysis at 30 min after MA is reached (LY30) as well as the novel measurements: SP, delta and the values from the velocity curve (TG, MRTG and TMRTG reflecting thrombin generation and MRL and TMRL reflecting clot lysis). The VC parameters used for all statistical analyses and figures were obtained from the MA version of calculation. For the sake of better comparison with other studies and potential future use by other investigators, reference intervals of VC parameters will be provided for both the G and MA version of calculation.

### Statistical analysis

Preliminary RIs were established for a citrate storage time of 60 min. If samples were measured in duplicates (as done for assessment of variance), the first result was used. Data distribution regarding normality was assessed by reviewing the histogram and the QQ plot as well as using the Anderson-Darling test or a symmetry test for Robust provided by the software (Reference Value Advisor 2.1 [28]). For all variables except for TG and TMRL, a robust method after Box-Cox transformation (Reference Value Advisor 2.1 [28]) was used. For TG, the RI was calculated with a parametric method after Box-Cox transformation and RI for TMRL was established by use of a parametric method of untransformed data respectively. Mean, median, minimum and maximum values as well as standard deviation was determined from untransformed data. Pooled variance was used to assess the percentage of total variation that occurred between the duplicates.

For all statistical comparisons, a Shapiro-Wilk test was done in advance to evaluate the assumption of normality. The impact of citrate storage times was assessed using a paired t-test for alpha, G, CI, TG, MRTG and TMRTG and a Wilcoxon test for R, K, MA, LY30, SP, delta, TMRL, MRL respectively. Because of the low number of cats in the group of high grade clumping the assessment of normality is limited so the influence of platelet clumps on the results was assessed by a nonparametric test (Mann-Whitney U test) for all variables. For all analyses, a *p*-value <0.05 was considered significant.

## Results

### Hematological analyses

The platelet count (PLT) estimated from the blood smear showed a thrombocytopenia (36–124 × 10^3^/μl) in five cats, four of which (PLT: 44–124 × 10^3^/μl) had marked aggregation on the blood smear so the count was considered falsely low. The remaining cat showing a thrombocytopenia (36 × 10^3^/μl) only had a moderate degree of platelet clumping. This cat did not show a thrombocytopenia on the CBC 1 week earlier and was clinically healthy without a history of bleeding so it was still included in the study.

Re-evaluation of the corrected hematocrit on day of sampling demonstrated no major abnormalities.

### Analyses of plasmatic hemostasis

Mean, median, range and standard deviation for TT, PT, aPTT, fibrinogen and D-dimers are shown in Table 1. Compared to the laboratory internal RIs which are also listed in Table 1, all values were considered normal denoting a physiological secondary hemostasis in all cats.

**Table 1:** Results of the traditional coagulation profile obtained from 21 cats

Variable	TT (sec)	PT (sec)	aPTT (sec)	Fibrinogen (g/l)	D-dimers (μg/ml)
Mean	15.37	10.8	11.92	1.46	0.18
Median	15.4	10.8	12	1.48	0.2
Range	14–16.9	10.1–12.8	10.8–13	1.04–1.7	0.09–0.26
SD	0.82	0.56	0.74	0.17	0.05
RI (laboratory-internal^a^)	13.4–19.1	9.9–12.2	10.8–14.0	0.6–2.2	0.0–0.3

*Abbreviations*: *SD* standard deviation, *RI* reference interval, *TT* thrombin time, *PT* prothrombin time, *aPTT* activated partial thromboplastin time, *sec* seconds

^a^For interpretation of the values, laboratory-internal reference intervals (RIs) established from 40 clinically healthy domestic shorthair cats using a robust method of untransformed data are additionally listed

### Preliminary RIs

One cat (1/21) had to be excluded because no TEG tracing could be obtained. This cat showed a marked aggregation of platelets on the blood smear and an estimated PLT count of 44 × 10^3^/μl. The routine coagulation parameters were unremarkable in this cat.

Preliminary RIs, CVs as well as the mean, median, the standard deviation, minimum and maximum for the investigated TEG variables of citrated whole blood samples stored for 60 min are shown in Table 2.

**Table 2:** Shows preliminary reference intervals (RI) for all investigated trombelastography (TEG) variables

Variable (unit)	R (min)	K (min)	alpha (degree)	MA (mm)	G (dyn/cm^2^)	CI (unitless)	LY30 (%)	SP (min)	delta (min)	TG^a^ (mm/ min)	MRTG^a^ (mm/ min)	TMRTG^a^ (min)	MRL^a^ (mm/min)	TMRL^a^ (min)
Number of cats (n)	20	19	20	20	20	19	20	20	20	20	20	20	19	19
RI	2.7–18.1	^b^ 0.8–3.9	27.6–75.2	18.5–62.5	1.2–8.2	^b^ −4.6 – 2.6	^c^	2.4–15.4	0.3–3.1	255.3–751.2	4.0–19.3	3.5–22.0	0.0–4.7	0.4–55.8
Minimum	2.8	1	26.1	18.2	1.1	−4.6	2.4	2.4	0.3	244.87	3.48	3.67	0.13	9.58
Maximum	18.7	3.6	74.9	61.4	8	2.5	15.8	15.8	2.9	735.09	19.28	22.5	4.37	56.08
Mean	7.6	2.3	57.6	46.3	4.6	−0.2	11.1	6.6	1.0	563.1	9.4	9.8	1.8	28.1
Median	6.6	2.3	58.1	47.3	4.5	0.1	6.6	5.8	0.9	588.5	8.5	8.8	1.8	22.4
SD	3.8	0.7	10.7	10.0	1.7	1.6	11.6	3.2	0.6	112.9	3.5	4.5	1.1	12.8
CV(%)	15.2	4.7	13.2	8.4	19.5	83.6	57.2	15.3	23.6	8.9	11.5	27.1	33.9	35.7

*Abbreviations*: *RI* reference interval, *min* minutes, *SD* standard deviation, *CV* coefficient of variation, *R* reaction time, *K* clotting time, *MA* maximum amplitude, *G* clot rigidity, *CI* coagulation index, *LY30* clot lysis at 30 min, *SP* split point, *TG* total thrombin generation, *MRTG* maximum rate of thrombus generation, *TMRTG* time to maximum rate of thrombus generation, *MRL* maximum rate of lysis, *TMRL* time to maximum rate of lysis

^a^Velocity curve variables (TG, MRTG, TMRTG, MRL, TMRL) were obtained as MA version

^b^Reference intervals are not considered valid according to the ASVCP (American Society of Veterinary Clinical Pathology) guidelines as number of animals <20

^c^A valid reference interval could not be calculated with any statistical method

**Fig. 3:**
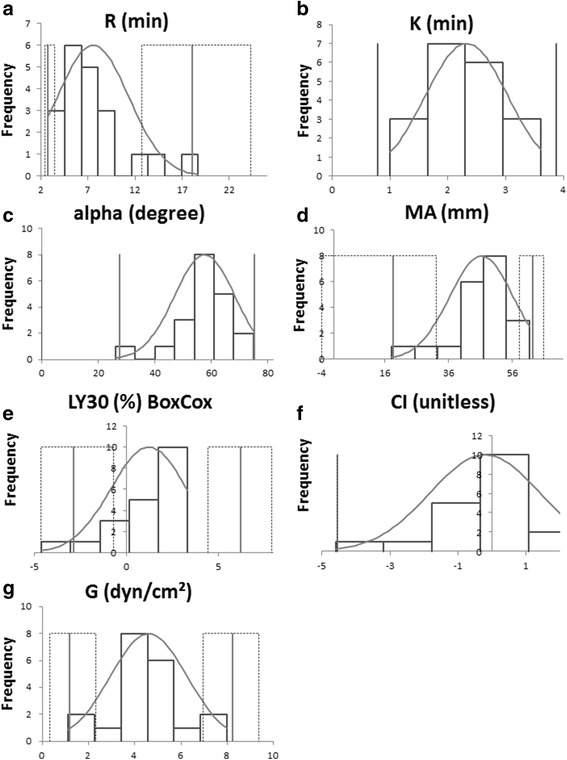
Histograms showing distribution of untransformed (all but LY30) data obtained for calculation of the reference interval of the traditional TEG. Data for measurement included the first results of all samples after 60 min of storage time. The solid grey line represents the upper and the lower limit of the reference interval. The small black dotted line is consistent with the 90% confidence interval. The light grey line indicates the Gaussian curve. Abbreviations: R = reaction time (**a**), K = clotting time (**b**), Alpha = alpha angle (**c**), MA = maximum amplitude (**d**), LY30 = lysis at 30 min (**e**), CI = coagulation index (**f**), G = clot rigidity (**g**)

**Fig. 4:**
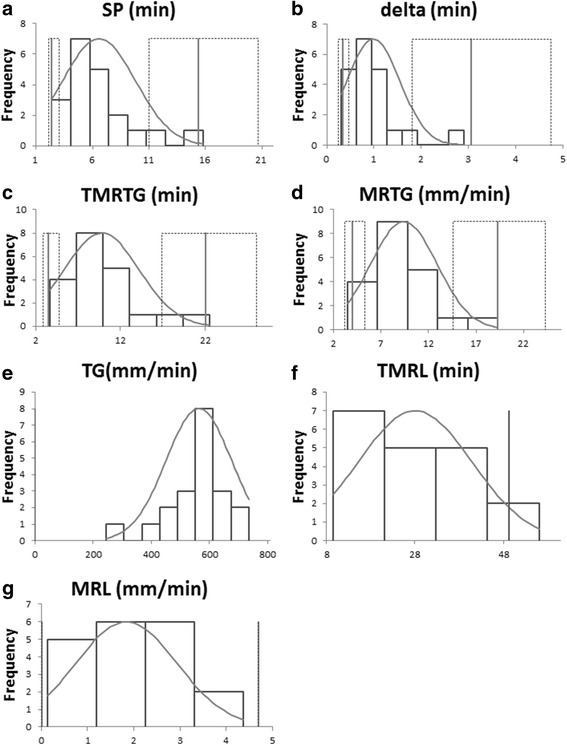
Histograms showing the distribution of untransformed data for calculation of reference intervals of the novel TEG variables SP, delta, TMRTG, MRTG, TMRL and MRL respectively. Data for measurement included the first results of all samples after 60 min of storage time. The solid grey line represents the upper and the lower limit of the reference interval. The small black dotted line is consistent with the 90% confidence interval. The light grey line indicates the Gaussian curve. Abbreviations: SP = split point (**a**), D = delta (**b**), TMRTG = time to maximum rate of thrombus generation (**c**), MRTG = maximum rate of thrombus generation (**d**), TG = thrombus generation (**e**), TMRL = time to maximum rate of lysis (**f**), MRL = maximum rate of lysis (**g**)

Histograms of the distribution of all evaluated variables after Box Cox transformation (LY30) or untransformed data (all but LY30) are shown in Figs. 3 and 4. In one cat (1/20), the CI and K value were not reported by the TEG software and in another cat (1/20) no results for MRL and TMRL were obtained. For LY30, data distribution did not allow the calculation of a reliable RI.

The CV was <20% for the variables R, K, alpha, MA, G, SP, TG and MRTG. For the other evaluated variables (CI, LY30, delta, TMRTG, MRL and TMRL) CVs were ranging between 23.6% (delta) and 83.6% (CI) respectively. Statistical analysis did not reveal a significant difference between the first and the second measurement performed after a citrate storage time of 60 min for all assessed variables.

### Impact of citrate storage time

**Fig. 5:**
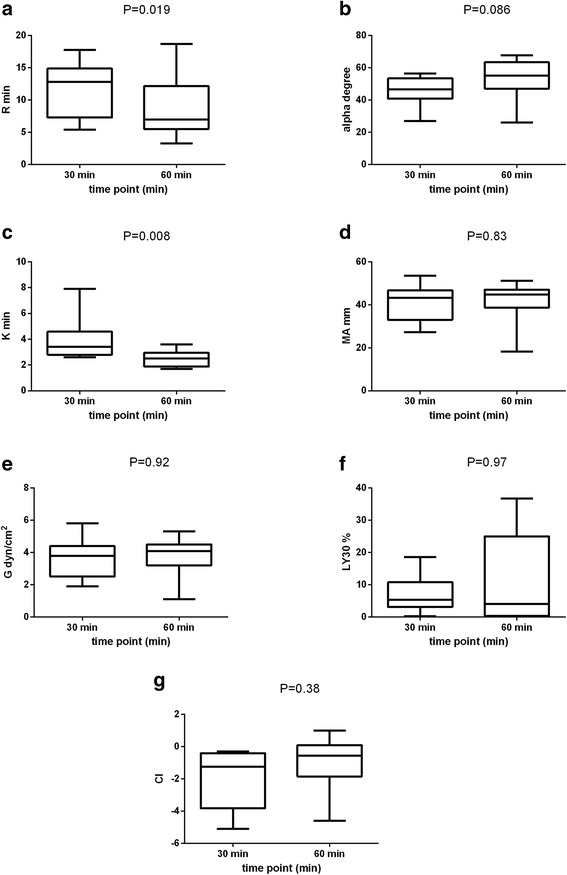
Effect of citrate storage time (30 min versus 60 min) on the traditional thrombelastographic (TEG) variables expressed as box and whisker diagrams. The central box represents values from the lower (25th) to upper (75th) percentile, while the middle line is consistent with the median. The horizontal line depicts the minimum and maximum values. The level of significance was set at *P* < 0.05. Abbreviations: R = reaction time (**a**), alpha degree (**b**), K = coagulation time (**c**), MA = maximum amplitude (**d**), G = clot rigidity (**e**), LY30 = lysis at 30 min (**f**), CI = coagulation index (**g**)

**Fig. 6:**
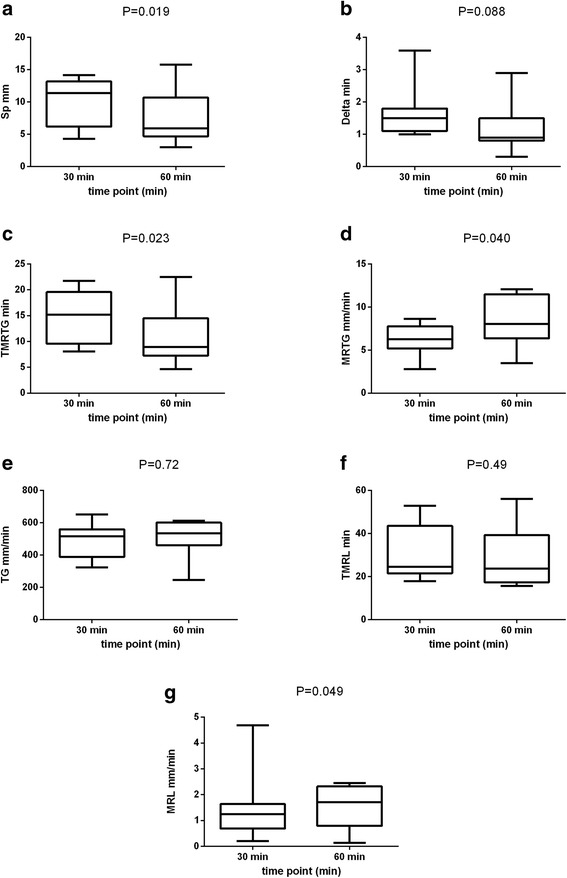
Effect of citrate storage time (30 min versus 60 min) on the novel thrombelastographic (TEG) variables expressed as box and whisker diagrams. The central box represents values from the lower (25th) to upper (75th) percentile, while the middle line is consistent with the median. The horizontal line depicts the minimum and maximum values. The level of significance was set at *P* < 0.05. Abbreviations: SP = split point (**a**), D = delta (**b**), TMRTG = time to maximum rate of thrombus generation (**c**), MRTG = maximum rate of thrombus generation (**d**), TG = thrombus generation (**e**), TMRL = time to maximum rate of lysis (**f**), MRL = maximum rate of lysis (**g**)

As seen in Figs. 5 and 6, citrate storage time had a significant impact on many traditional and novel TEG variables. When evaluating the traditional TEG variables (Fig. 5), prolonged citrate storage time (60 versus 30 min) induced a significant decrease in median values of R (7 min. vs. 12.8 min., *P* = 0.019) and K (2.5 min. vs. 3.4 min., *P* = 0.008) respectively. Interestingly, a marked increase in inter-individual variation of LY30 was noted with increasing citrate storage time. Regarding the novel variables (Fig. 6), the TMRTG was significantly shorter (8.92 min. vs. 15.17 min., *P* = 0.023), the MRTG was significantly larger (8.03 mm/min vs. 6.26 mm/min, *P* = 0.04) and the SP was significantly smaller (5.9 min. vs. 11.4 min., P = 0.019) after 60 min when compared to results after 30 min of storage. Overall, these results reflect the development of a more hypercoagulable TEG tracing with increasing citrate storage time. For the variables of clot lysis, only MRL was significantly influenced by citrate storage time with values being higher at 60 min compared to 30 min (1.71 mm/min vs. 1.25 mm/min, *P* = 0.049). In contrast, there was no significant impact of citrate storage time on the other investigated TEG variables (alpha, MA, G, CI, delta, LY30, TG, TMRL).

**Fig. 7:**
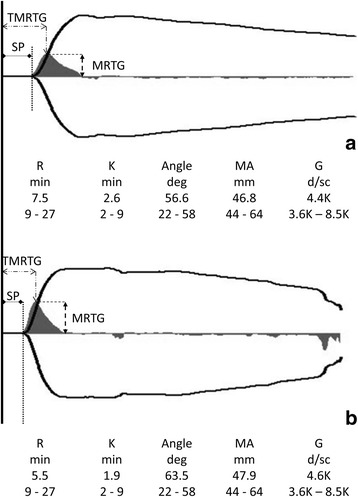
Exemplarily illustration of the effect of citrate storage on the conventional thrombelastographic (TEG) tracing and the velocity curve (solid grey curve) in one cat included in the study. **a**: Analysis after 30 min, **b**: Analysis after 60 min. Note the shortening of the R-value and the K-value as well as the increase of MRTG and shortening of TMRTG consistent with a “hypercoagulable” TEG after increased incubation time. Abbreviations: TMRTG = time to maximum rate of thrombus generation, SP = split point, MRTG = maximum rate of thrombus generation.

Examples of two TEG tracings performed in a cat after a citrate storage time of 30 and 60 min respectively is depicted in Fig. 7 clearly demonstrating the decrease in R, K, SP and TMRTG as well as an increase in MRTG.

### Impact of platelet clumping

No statistically significant differences could be found between the group of cats classified as having low grade clumping and those cats classified as showing high grade platelet clumping. Table 3 provides the range and median of the different groups as well as the *p*-value between these two groups.

**Table 3:** Comparison of the TEG results of different groups of cats categorized by the grade of platelet clumping

Group of cats	Low grade of clumping			High grade of clumping			*p*-value
Variable (unit)	Number of cats	Range	Median	Number of cats	Range	Median	
R (sec)	13	3.3–18.7	6.5	5	5.8–9.6	8.1	0.29
K (sec)	12	1.4–3.4	2.4	5	1.7–3.4	2.3	0.67
Alpha (°)	13	26.1–70	57.8	5	49.3–66.4	58.3	0.94
MA (mm)	13	18.2–61.4	46.6	5	42.1–60.9	52.6	0.12
G (dyn/cm^2^)	13	1.1–8	4.4	5	3.6–7.8	5.5	0.11
LY30 (%)	13	0–36.8	5.3	5	1.2–27.6	7.9	0.92
CI (unitless)	12	−2.3 – 2.5	−0.2	5	−1.5 – 2.1	0.3	0.34
SP (min)	13	3–15.8	5.7	5	5.2–8.6	7	0.25
Delta (min)	13	0.3–2.9	0.8	5	0.6–1.1	1	0.52
TMRTG (min)	13	4.7–22.5	8.8	5	7.1–11.9	10.3	0.62
MRTG (mm/min)	13	3.5–14.7	8.1	5	6.2–12.2	9.6	0.63
TG (mm/min)	13	244.9–735.1	565	5	517.5–734.3	636.3	0.12
TMRL (min)	12	15.7–56.1	21.2	5	22.3–46.3	34.1	0.08
MRL (mm/min)	12	0.1–4.4	1.8	5	0.3–2.5	1.4	0.59

*Abbreviations*: *SD* standard deviation, *R* reaction time, *K* clotting time, *MA* maximum amplitude, *G* clot rigidity, *CI* coagulation index, *LY30* clot lysis at 30 min, *SP* split point, *TG* total thrombin generation, *MRTG* maximum rate of thrombus generation, *TMRTG* time to maximum rate of thrombus generation, *MRL* maximum rate of lysis, *TMRL* time to maximum rate of lysis

## Discussion

To the authors’ knowledge, this is the first study evaluating novel TEG variables such as SP, delta and the VC variables for cats as well as the impact of storage time on feline TEG tracings.

There is no definite evidence why one cat (excluded from the study) only showed flat lines drawing the conclusion that no clot could be detected by the analyzer. Because the routine plasmatic coagulation parameters were unremarkable in this cat, a coagulation disorder seems unlikely. Relating to the TEG being a test of global hemostasis, a platelet origin might be the reason of undetectable clots in this cat. In fact, this cat showed marked aggregation of platelets on the blood smear, but since five different cats also showed moderate to marked platelet aggregation on the blood smear and TEG tracings could still be obtained, an aggregation of platelet might not be the only reason. Further studies should be obtained to verify this observation.

Our study demonstrated a significant impact of citrate storage time on several traditional and novel TEG variables. Consequently, RI’s are also influenced by the citrate storage time and thus should be established for any citrate storage time intended to be used in the laboratory. However, when using the RI, the relatively high CVs of many TEG variables have to be taken into account.

One limitation of our study is the small number of cats included so that potential significant effects might have been missed. Moreover, the establishment of RIs is hampered by the small sample size and RIs determined here have to be considered as preliminary values. As recommended by the American Society of Veterinary Clinical Pathology (ASVCP) guidelines for reference samples 20 ≤ x < 40, a robust method was mostly used for the establishment [29]. Furthermore, these guidelines suggest that reference intervals should not be obtained when less than 20 samples are available [29] as was the case for K, CI, MRL and TMRL. For reference samples 10 ≤ x < 20, the ASVCP guidelines recommend to report histograms (Figs. 3 and 4) as well as mean and median values (Table 2) to help facilitate interpretation of the values.

A direct comparison of RIs obtained in this study using a storage time of 60 min to the results of other studies cannot be made without serious limitations since other studies used a different citrate storage time (i.e., 30 min) [19, 21] or different modes of activation [19, 21] which also can affect TEG variables. With regard to the velocity variables, no other study evaluating the VC in feline thrombelastography was found. For better comparability, Table 4 provides the RIs of both the MA version and G version of the velocity curve variables, which are slightly different since the curves (MA version and G version) are minimally different.

**Table 4:** Comparison of the velocity curve reference intervals based on the maximum amplitude or clot rigidity

MA version		G version	
Variable (unit)	RI	Variable (unit)	RI
TMRTG (min)	3.5–22.0	TMRTGG (min)	4.1–23.0
MRTG (mm/min)	4.0–19.3	MRTGG (dsc)	2.1–11.9
TG (mm/min)	255.3–751.2	TGG (dsc)	138.8–828.5
TMRL (min)	0.4–55.8	TMRLG (min)	0.0–55.9
MRL (mm/min)	0.0–4.7	MRLG (dsc)	−1.6 – 7.7

For each variable, in both versions the same statistical method was used. G version variables show an appended “G”

*Abbreviations*: *MA* maximum amplitude, *G* clot rigidity, *RI* reference interval, *TMRTG(G)* time to maximum rate of thrombus generation, *MRTG(G)* maximum rate of thrombus generation, *TG(G)* (total) thrombus generation, *TMRL(G)* time to maximum rate of lysis, *MRL(G)* maximum rate of lysis, *min* minute, *mm* millimeter, *dsc* dyn/cm^2^/s

Regarding the stability of citrated whole blood, our study shows that longer storage times induce a more hypercoagulable TEG tracing which is not only represented by a shorter R, K and SP but also can be seen on the velocity curve by reduced TMRTG and MRTG values.

The R value reflects the time from sample placement in the cuvette until an amplitude of 2 mm is reached on the TEG tracing. It is dependent on plasma clotting factors and circulating inhibitor activity and represents the initial fibrin formation rate [1]. The K value is a measurement from the point where 2 mm of amplitude is reached (R time) to the point of 20 mm amplitude. This time reflects the buildup and cross linking of fibrin, which is related to clotting factors such as factor II and VII, fibrinogen and platelets count and function [1].

Furthermore, alpha and delta show a non-significant trend towards hypercoagulability at 60 min. Alpha (°) is the angle formed by the TEG tracing between the R and the K value and reflects the speed of clot formation [1]. As alpha and MRTG reflect a similar item, a similarly significant change could have been expected between the time points. However, alpha was not significantly altered while MRTG was significantly affected by different storage times. A possible explanation for this discrepancy could be their different mode of change during the coagulation process as described previously for humans [7]: compared to alpha which is variable of the traditional curve and shows a non-exponential behavior, MRTG as a parameter derived from the velocity curve changes in an exponential and thus markedly pronounced way.

A similar effect was noticed here for TMRTG which in previous studies in human medicine has shown a linear correlation with delta [6] so that theoretically a similar impact of storage on both variables would have been expected. However, the citrate storage time only had a significant impact on the VC parameter TMRTG increasing exponentially but not on delta derived from the traditional TEG curve changing in a non-exponential mode. In conclusion, some VC variables (TMRTG, MRTG) might be a more sensitive marker of changes in coagulation activity like hypercoagulability than traditional TEG values. This hypothesis however, further has to be substantiated in future clinical studies.

A previous investigation in critically ill patients could further demonstrate a linear relationship between delta and TMRTG reflecting thrombin generation [6]. In our study evaluating the impact of citrate storage time in TEG variables, the behavior of TMRTG resembled the findings observed for R more closely than those observed for delta. It thus could be hypothesized, that there is an even closer relationship between TMRTG and R than there is between TMRTG and delta. Reasons contributing to this difference in the current study compared to Gonzalez et al. [6] might be the different species, the different storage time, the different mode of activation and the prophylactic therapy with heparin potentially influencing the values as R partially depends on the activity of circulating inhibitors [1]. Further studies are needed to evaluate the relationship amongst this threesome (TMRTG, R and delta).

Regarding TG, previously investigated in humans as a marker of thrombin generation [4], the absolute amount of thrombus generated (TG) in our study in cats was interestingly not altered by prolonged citrate storage times. In conjunction with the changes in TMRTG and MRTG, citrate storage time apparently rather affects the speed of clot formation and thrombin generation (as TMRTG is believed to be a marker of thrombin generation [6]) than the total amount of thrombin generated.

In agreement with our findings in cats, significant differences with a tendency towards hypercoagulability with longer storage time have been observed in dogs [3] and horses [17]. In contrast to that, another study in horses reported sample (citrated whole blood) stability at room temperature for up to 20 h prior to TEG measurement, however, measurements were made after 2 and 19 to 48 h of citrate storage time so that changes prior 2 h of storage might have been missed [18].

Despite from an incomplete inhibition of thrombin formation during storage [3], repeated sampling from a single tube could function as a “contact activator” for the coagulation cascade via the pipette surface which in humans has been shown to alter TEG results [16]. The factor of repeated sampling has not been determined in our study but in other veterinary studies which used separate tubes for different time points to avoid repeated pipetting and still showed significant differences with a tendency towards hypercoagulability [3, 17]. Hypercoagulable TEG tracings with prolonged storage times might thus be resulting from different in vitro blood stability of different mammalian species [17].

## Conclusion

Storage of citrated whole blood used for TEG analysis in cats has significant effects on both traditional and VC variables with a trend towards hypercoagulability with prolonged storage times. By that, some velocity curve variables (explicitly TMRTG, MRTG) might help in the early detection of changes in coagulation activity for example hypercoagulable states.

Because of the effect of citrate storage times on TEG analysis, RIs can only be used for a specific storage time which has to be established in each laboratory.

## Abbreviations

aPTT: activated partial thromboplastin time
CI: coagulation index
cm: centimeter
CV: coefficients o variation
dsc: dyn/cm^2^/s
G: clot rigidity
K: clotting time
LY30: lysis at 30 min after MA is reached
MA: maximum amplitude
min: minute
mm: millimeter
MRL: maximum rate of lysis
MRTG: maximum rate of thrombus generation
PT: prothrombin time
R: reaction time
RIs: reference intervals
sec: second
SP: split point
TEG: thrombelastography
TG: (total) thrombus generation
TMRL: time to maximum rate of lysis
TMRTG: time to maximum rate of thrombus generation
TT: thrombin time
VC: velocity curve
